# Expression Level of IL-6 Secreted by Bone Marrow Stromal Cells in Mice with Aplastic Anemia

**DOI:** 10.1155/2013/986219

**Published:** 2013-06-18

**Authors:** Yong Feng Chen, Zhong Min Wu, Cong Xie, Shi Bai, Li Dong Zhao

**Affiliations:** ^1^Department of Basic Medical Sciences, School of Medicine of Taizhou University, Taizhou 318000, China; ^2^General Affairs Office of Taizhou University, Taizhou 318000, China

## Abstract

Parasecretion of the hematopoietic cytokines is considered as one of the mechanisms account for bone marrow hematopoiesis disorder. In this study, the level of IL-6 secreted by bone marrow stromal cells from a mouse model of aplastic anemia was analyzed. The aplastic anemia mouse model was established with cyclophosphamide in combination with chloramphenicol and ^60^Co**γ** radiation. The impairment of bone marrow hematopoiesis induced by irradiation and chemotherapeutic drugs was subsequently characterized by peripheral blood cell count, pathomorphological changes, and apoptosis rate. Furthermore, the *in vitro* proliferation of bone marrow stromal cells (BMSC) and the IL-6 secretion levels of BMSC were analyzed. In our model of aplastic anemia, the number of peripheral blood cells and bone marrow cells (BMC) were notably decreased, and the apoptosis rate of BMC increased. Furthermore, the proliferation of BMSC was obviously impeded while the IL-6 secretion levels of BMSC significantly increased. The findings of our study suggested that the IL-6 secretion level may be enhanced to some extent by the induction of aplastic anemia caused by irradiation and chemotherapeutic drugs and that the abnormal level of IL-6 might probably interfere with the stability of the bone marrow hematopoietic microenvironment.

## 1. Introduction

Bone marrow is the major hematopoietic organ. Bone marrow hematopoietic tissues are highly sensitive to toxins, including radiation and chemotherapeutics, which may induce myelosuppression. The cytoactivities of primitive hematopoietic cells may, therefore, be depressed, and the number of peripheral blood cells may decrease substantially. In addition, severe myelosuppression may cause infections, inflammation, and bleeding [1, 2]. 

It is generally accepted that maintenance of normal hematopoiesis relies on the stabilization of the hematopoietic microenvironment. As the major component of this microenvironment, hematopoietic cytokines play a key role in the regulation of hematopoiesis, controlling the proliferation, differentiation, and maturation of primitive hematopoietic cells [3, 4]. IL-6 is a kind of cytokine with a wide range of biological activities, produced mainly by T and B lymphocytes, monocytes, fibroblasts, and so forth [5, 6]. Recent studies showed that IL-6 involved not only in the progress of the stress reaction, autoimmune, and neoplastic diseases of the body but also in regulation of the proliferation and differentiation of primitive hematopoietic cells, and it is considered as a permissive factor of primitive hematopoiesis [7]. However, IL-6 is also a proinflammatory factor. Of all the cytokines, IL-6 is the first one found to be associated with the pathogenesis of the disease, due to its close relation with a variety of diseases under overexpression [8]. 

In the present study, a mouse model of aplastic anemia was established by using cyclophosphamide in combination with chloramphenicol and ^60^Co*γ* radiation. After modeling, the *in vitro* proliferation of BMSC was tested by MTT assay, and the IL-6 secretion levels of BMSC were analyzed. This study gives new insights into the hematopoiesis regulating properties of IL-6 in mice with aplastic anemia.

## 2. Materials and Methods

The procedures of our experiment are in compliance with the Principles of Laboratory Animal Care. All procedures were approved by the Animal Care and Use Committee of Taizhou University.

### 2.1. Reagents

Cyclophosphamide injections were obtained from the Shanghai Hualian Pharmaceutical Co., Ltd. Chloramphenicol injections were made by Southwest Pharmaceutical Stock Co., Ltd. IMDM tissue culture media and IL-6 ELISA kits were purchased from the Jingmei Company (Chengdu, China). The TUNEL kit was purchased from the Boster Company (Wuhan, China).

### 2.2. Equipment

The fully automatic blood cell analyzer (HS-18) was made in Italy; the inverted phase contrast microscope by the Chong Qing Optical Instrument Factory (China); the CO_2_ incubator (MCD-15A) in Japan; and the fully-automatic ELISA meter by the Thermo Company (USA).

### 2.3. Animals

Healthy BALB/c male mice, weighing 18–22 g, aged 6–8 weeks, were provided by the Experimental Animal Center of Chengdu University of Traditional Chinese Medicine. Animals were housed in a warm, quiet environment with free access to food and water. All mice were acclimatized for 1 week before the beginning of the experiments.

### 2.4. Radiation

 The radiation source (^60^Co*γ*) was provided by Sichuan Academy of Agricultural Sciences.

### 2.5. Induction of Aplastic Anemia

 The mice were randomly divided into normal control and aplastic anemia groups. The aplastic anemia model was made according to Zhang's method [9]. Briefly, the mice were irradiated with 2.0 Gy ^60^Co*γ* and then treated with daily intraperitoneal injections of cyclophosphamide at 40 mg/kg/day and chloramphenicol at 50 mg/kg/day for the next three days. 

### 2.6. Peripheral Blood Cells and BMC Count

On day 7 after modeling, 20 *μ*L blood was taken from the orbital vein of the mouse and diluted with the manufacturer recommended diluent before analysis with the HS-18 fully automatic blood cell analyzer. Eight mice from each group were sacrificed, and the femurs of each mouse were removed. The BMC suspension was prepared according to Zhang's method [9], and the number of BMC was counted.

### 2.7. Pathomorphological Change Analysis

On day 7, two mice from each group were randomly chosen, the femurs and spleens were removed, fixed in 4% neutral formalin, decalcified, and desiccated, and embedded in paraffin, and then semithin sections were cut. After hematoxylin-eosin staining, the pathomorphological changes of bone marrow and spleen were observed with a light microscope.

### 2.8. BMC Apoptosis Rate Analysis

On day 7, BMC smears were prepared, and BMC apoptosis was analyzed by TUNEL, according to the specifications of the TUNEL kit. 5 microscopic fields of each slide were randomly chosen and observed under microscope, and the apoptosis ratio was calculated.

### 2.9. IL-6 Analysis

On the 4th, 7th, and 10th days after modeling, BMC suspensions were prepared as described above then adjusted to a concentration of 1 × 10^6^ cells/mL. BMC were cultured in IMDM supplemented with 15% fetal calf serum at 37°C in 5% CO_2_ and saturated humidity. After 48 h of cultivation, culture supernatants were harvested and stored at −20°C for IL-6 analysis. The proliferation of the bone marrow stromal cells was evaluated by MTT method [10]. MTT was added to every well, and the culture medium was put back to the incubator. 4 h later, the MTT was removed and the cells were washed by PBS twice. 100 *μ*L of isopropyl alcohol was added to split the cells. Then, the enzyme-linked immunosorbent assay was used at the 570 nm to detect the *A* value of every well. IL-6 level was also measured with ELISA.

### 2.10. Statistical Analysis

All data were expressed as mean ± standard deviation $\left(\overset{-}{x}\pm s\right),$ and the *t*-test was used to evaluate the difference between groups, with *P* < 0.05 considered to be statistically significant.

## 3. Results

### 3.1. Peripheral Blood and BMC Count

**Figure 1:**
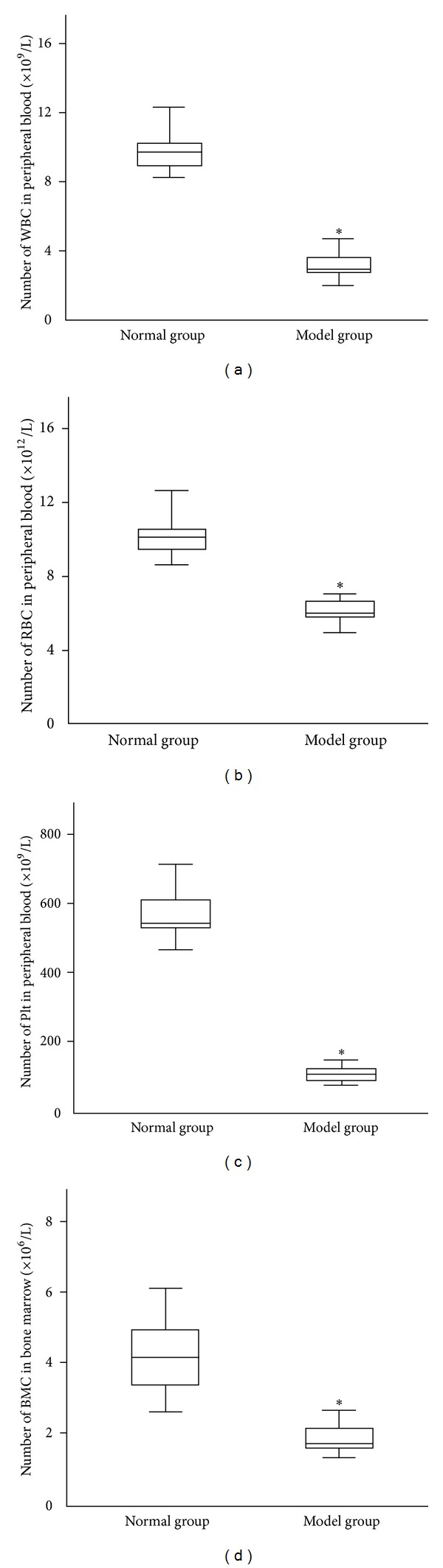
Peripheral blood and BMC count in normal and model mice ($\overset{-}{x}\pm \text{s}$, *n* = 8). (a) WBC, (b) RBC, (c) Plt, and (d) BMC. After modeling, the number of WBC, RBC, Plt, and BMC in model was significantly decreased. Statistical analyses of the data were performed using one-way ANOVA, followed by Scheffe's *post hoc* test (*P < 0.05, compared with normal group).

As measured by the fully automatic blood cell analyzer, the number of peripheral blood cells in the aplastic anemia mouse model was notably decreased (*P* < 0.05). Besides, the number of BMC was markedly decreased (*P* < 0.05). These data indicated that the aplastic anemia model had been successfully established (Figure 1). 

### 3.2. Pathomorphological Change of Bone Marrow and Spleen

**Figure 2:**
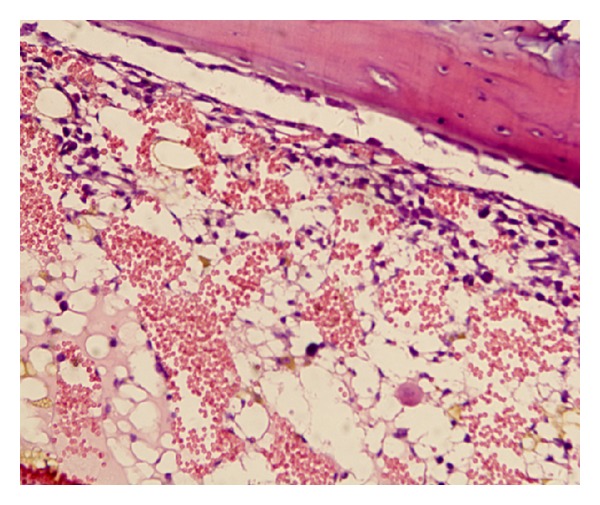
Pathomorphological change of bone marrow. The number of hematogenous cells decreased and fat cells increased markedly in the bone marrow of model mouse (100×).

**Figure 3:**
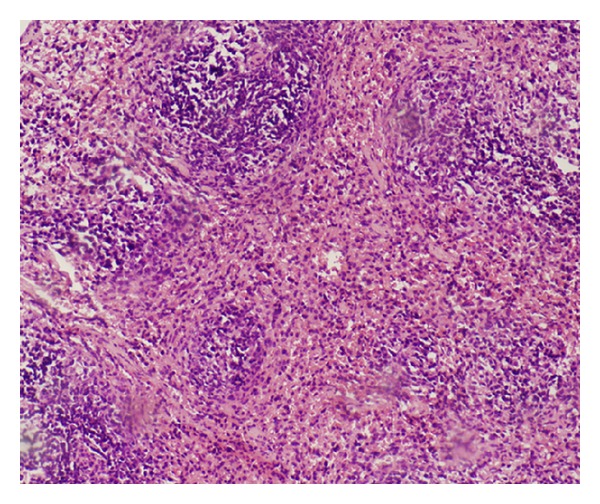
Pathomorphological change of spleen. After modeling, the spleen atrophied and the splenic corpuscle shrank. Spleen blood sinuses dilated, and the hematopoietic focus disappeared (100×).

The morphology of the bone marrow tissue had changed obviously after modeling. In our model, the amount of hematopoietic tissue, as well as the number of hematogenous cells in bone marrow, was obviously reduced. The number of lymphocytes, fat cells, and other nonhematogenous cells, however, increased markedly. These changes were accompanied by interstitial edema and blood sinus expansion (Figure 2). The spleen atrophied, the splenic corpuscle shrank or disappeared, and its structure was clearly compromised. Spleen blood sinuses dilated, hematopoietic focus disappeared, and megakaryocytes decreased or were absent (Figure 3).

### 3.3. BMC Apoptosis Rate Analysis

**Figure 4:**
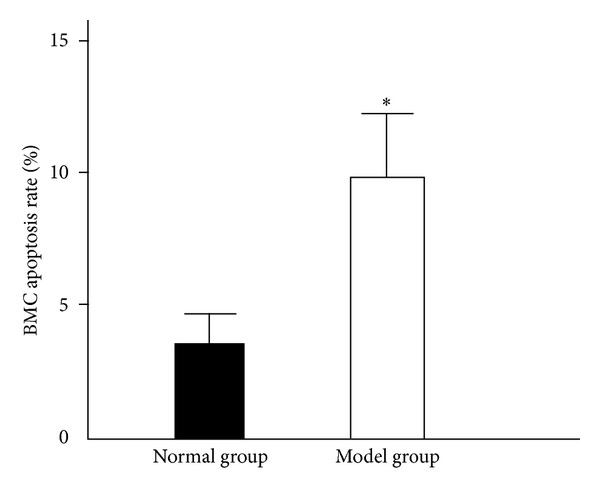
Apoptosis rate of bone marrow nucleated cells ($\overset{-}{x}\pm \text{s}$, *n* = 8). The apoptosis rate of BMC was significantly enhanced after modeling. Statistical analyses of the data were performed using one-way ANOVA, followed by Scheffe's *post hoc* test (*P < 0.05, compared with normal group).

**Figure 5:**
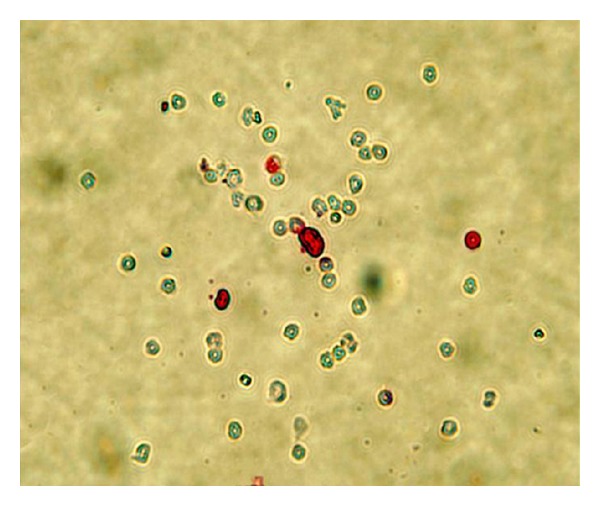
BMC apoptosis analysis by TUNEL. Nuclei of the apoptotic BMC were stained red with fast red while nuclei of nonapoptotic BMC were stained blue with hematoxylin (200×).

 The apoptosis rate of BMC from the aplastic anemia group (9.75 ± 2.43%) was significantly higher than that of normal controls (3.63 ± 1.06%; Figure 4) (*P* < 0.05). Nuclei of the apoptotic BMC were stained red with fast red while nuclei of nonapoptotic BMC were stained blue with hematoxylin (Figure 5).

### 3.4. IL-6 Level Analysis

**Figure 6:**
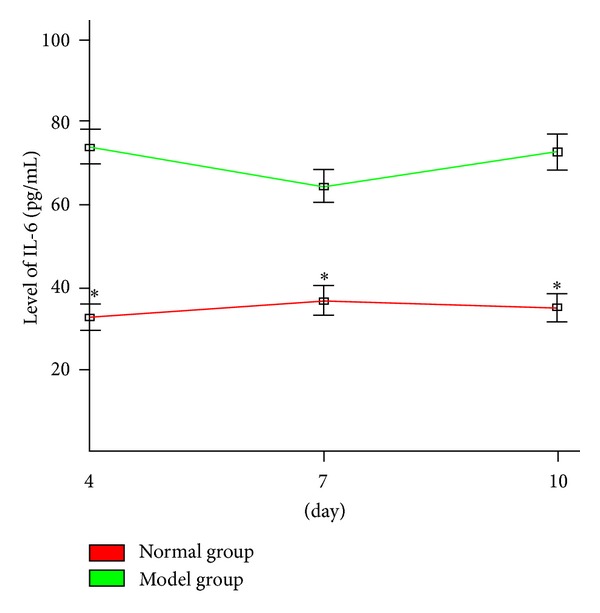
IL-6 expression level in the culture supernatant of BMSC ($\overset{-}{x}\pm s$, *n* = 6). The level of IL-6 in cultured supernatants of BMSC from the anemia group was significantly higher than that of the normal group. Statistical analyses of the data were performed using one-way ANOVA, followed by Scheffe's *post hoc* test (*P < 0.05, compared with normal group).

Figure 6 showed that at the three time points (4th day, 7th day, and 10th day), the level of IL-6 in cultured supernatants of BMSC from the anemia group was significantly higher than that of the normal group (*P* < 0.05).

### 3.5. Proliferation of BMSC

**Figure 7:**
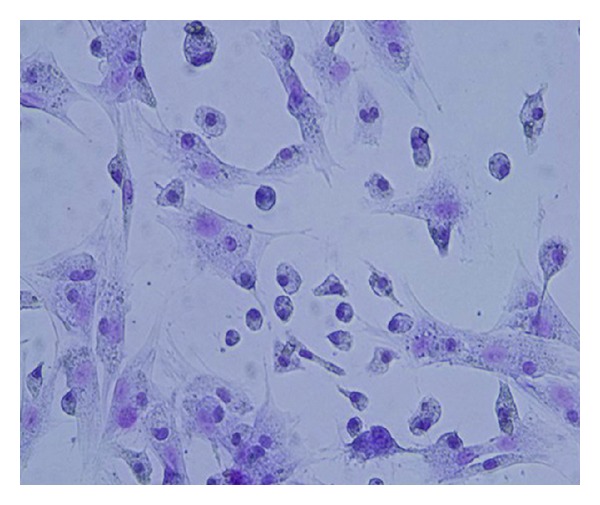
Bone marrow stromal cells stained with hematoxylin and eosin (200×).

**Figure 8:**
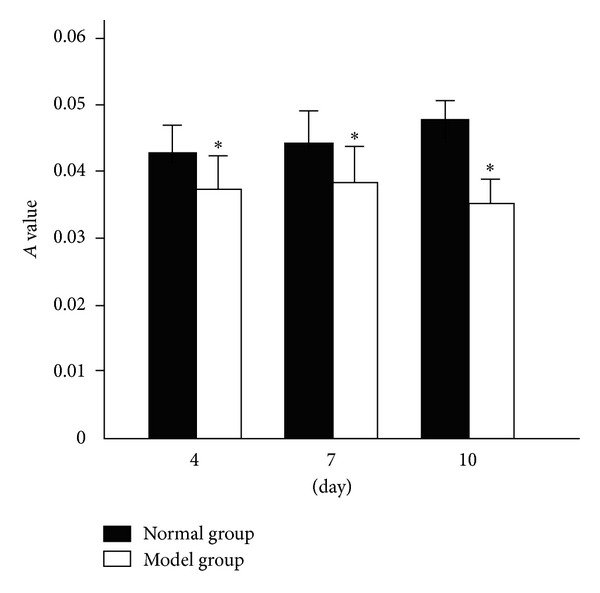
A value of bone marrow stromal cells ($\overset{-}{x}\pm s$, *n* = 6). Statistical analyses of the data were performed using one-way ANOVA, followed by Scheffe's *post hoc* test. The level of statistical significance was set at *P* < 0.05. *P < 0.05, compared with normal group.

BMSC displayed long fusiform shape under invert phase contrast microscope cultured more that 2 days (Figure 7). The A value of model group at the three time points (4th day, 7th day, and 10th day) was significantly lower than that of normal group (*P* < 0.05) (Figure 8).

## 4. Discussion

IL-6 is a frequently studied cytokine that has a wide range of biological activities. It has many target cells including macrophages, liver cells, stationary T cells, activated B cells, and plasma cells. It mainly functions in an autocrine or paracrine manner with very complex biological effects. It can play important roles in the acute phase of the immune response, hematopoietic regulation, and other processes. Recent studies also found that IL-6 can stimulate the formation of myeloid, erythroid, megakaryocyte, and macrophage cloning, in combination with EPO, IL-3, and other hematopoietic cytokines [11]. As IL-6 has important functions in proliferation, differentiation, and maturation of early HSC, it is considered as a permissive factor of primitive hematopoiesis [7]. 

Defective bone marrow stroma, or microenvironment, has been proposed as one of several mechanisms to account for bone marrow failure. This could involve defects in positive- or negative-acting hemopoietic regulator expression by stroma or alteration of normal stroma-stem cell interactions [12]. As the permissive factor of primitive hematopoiesis, the role of IL-6 in the bone marrow hematopoiesis disorder has been subject of intense attention in recent years. So far, the precise mechanism of IL-6 in hematopoiesis regulation still remains largely unclear. In the present study, a mouse model of aplastic anemia was established by using cyclophosphamide in combination with chloramphenicol and ^60^Co*γ* radiation. After modeling, the peripheral blood cells and BMC were significantly reduced and the apoptosis of BMC was obviously increased. Decreased hematogenous cells and increased fat cells in the bone marrow of the mouse model were easily observable with a microscope. Furthermore, the spleen shrank, the splenic sinus was enlarged, and the hemopoietic focus were evidently reduced. These results demonstrated that the bone marrow hematopoietic function was severely injured, and the mice model was successfully constructed. 

To investigate the level of IL-6 secreted by bone marrow stromal cells from the mouse model of aplastic anemia,in the present study, *in vitro* cultivation of bone marrow stromal cells was employed, and the IL-6 level was measured with ELISA. The result showed that at the three time points (4th day, 7th day, and 10th day), the level of IL-6 in cultured supernatants of BMSC from the anemia group was significantly higher than that of the normal group, which indicated that the IL-6 secretion level may be enhanced to some extent by the induction of aplastic anemia caused by irradiation and chemotherapeutic drugs. As a cytokine with a wide range of biological activities, IL-6 has very complex functions. IL-6 fulfills two-way functions of anti-inflammatory and proinflammatory. It is a key component of the inflammatory mediator network, with an important role in the inflammatory response. As an anti-inflammatory cytokine, IL-6 counteracts the damaging effects of proinflammation cytokines and protects the body. However, IL-6 is also a proinflammatory factor and, when overexpressed, can lead to a variety of diseases. Significantly elevated IL-6 levels were detected in the serum of rheumatoid arthritis, diabetes, acute pancreatitis, HIV, and cancer patients [13–16]. 

Herodin et al. reported that in radiation-induced bone marrow depressed baboons, a substantial precocious and transient IL-6 blood release was detected within hours after irradiation. The origin of the precocious transient IL-6 peak observed just after irradiation was not clear. It was supposed that the increase of IL-6 may be caused by a more precocious IL-1 and/or TNF release [17]. In a clinical experiment, Fu et al. found that the pretreatment level of IL-6 in serum and bone marrow of 37 aplastic anemia patients were slightly higher than that of normal control, but there was no significant difference between the aplastic anemia group and the normal control group. Fu et al. supposed that the change of IL-6 level in aplastic anemia patients may be due to an association with inflammatory response, and IL-6 may have an effect of immunoregulation and mediate induction in the destruction pathology of bone marrow cells, but the concrete mechanism still needs to be elucidated [18]. In our study, we noticed that in the anemia group, with the increase of IL-6 level, the proliferation of the BMSC was obviously impeded. This result indicated that high level of IL-6 might be harmful to the proliferation of bone marrow stromal cells and might interfere with the stability of the bone marrow hematopoietic microenvironment. As the main component of the bone marrow hematopoietic microenvironment, BMSC adhere to hematopoietic stem cells, support and regulate the internal environment for the settling, differentiation, proliferation, and maturation of the primitive hematopoietic cells, and secrete a variety of cytokines to regulate hematopoiesis; therefore, defects of the bone marrow stromal cells can cause hematopoietic disorders of the bone marrow [19, 20]. 

## 5. Conclusion

The results of our study suggested that the IL-6 secretion level may be enhanced to some extent by the induction of aplastic anemia caused by irradiation and chemotherapeutic drugs and that the abnormal level of IL-6 might probably interfere with the stability of the bone marrow hematopoietic microenvironment. Currently, the role of IL-6 in the regulation of hematopoiesis process and the specific mechanism remain unclear, so further investigations are needed.
